# Stylometry and forensic science: A literature review

**DOI:** 10.1016/j.fsisyn.2024.100481

**Published:** 2024-06-11

**Authors:** Valentina Cammarota, Silvia Bozza, Claude-Alain Roten, Franco Taroni

**Affiliations:** a School of Criminal Justice, University of Lausanne, Lausanne, Switzerland; b Department of Economics, Ca’ Foscari University of Venice, Venice, Italy; c OrphAnalytics SA, Vevey, Switzerland

**Keywords:** Forensic stylistics, Stylometry, Authorship attribution

## Abstract

The article focuses on a careful description of literature on stylometry and on its potential use in forensic science. The state of the art of stylometry is summarized to illustrate the history and the scientific foundation of this discipline. However, the study conducted reveals that there are still some key unresolved aspects that require a response from the academic world. The paper introduces the readers to those issues that need to be tackled for stylometry to be accepted as a forensic discipline. In particular, a coherent probabilistic procedure to assess the probative value of the results obtained through this methodology is largely absent. This gap should be filled properly by applying criteria recommended by international organizations such as the European Network of Forensic Science Institutes. Solutions do exist and will allow a better integration of stylometry in forensic science, favouring the acceptance of this scientific technical method in judicial proceedings.

## 1 Introduction

Over the last decade, there has been a growing interest in the statistical analysis of writing style, otherwise known as “stylometry” [1]. Central to the entire discipline is the idea that everyone has a different writing style, and although it varies over time, stylometry offer extremely valuable information for addressing authorship issues [2,3]. Style would thus represent a so-called and perceived identification means for forensic purposes [4].

Document analysis in forensic science is usually based on the study of a person's handwriting, or on the physico-chemical analysis of the support (generally, paper), the scripting instrument and/or possible printing characteristics [5]. The questions that arise in such a field may concern the origin, production, integrity or legitimacy of the written document [6].

For a long time, the experience of the mandating expert, upon which their opinions were based, represented the main criteria for a scientific report conclusion. Then, quantification tools were favoured for the description and, consequently, the evaluation of a selected list of features con-sidered of relevance for the characterization of the questioned document. This allows not to limit the expert's conclusions mainly to his personal experience [7]. A recent synthesis of the evolution of the state of the art in the field of document and signatures analyses towards quantification is provided by Gaborini [8] and Linden [9,10], respectively.

Preprint submitted to Elsevier May 30, 2024

The ever-growing interest in features description through quantification makes stylometry a promising technique to be adopted in forensic science. Stylometry, a sub-category of the forensic linguistic [[11], [12], [13]], is characterized by descriptive statistical studies of an author's writing style. A text is composed of words (semantic elements), which are organized in sentences (syntax). As the order of the nitrogenous bases determines one's genetic code, the syntax determines the author's style [14]. Textual authorship description takes advantage of discriminating stylistic features [15,16], typically chosen unconsciously by the writer of a text [17].

The contribution of forensic linguistics - a term coined by Svartvik in 1968 [12] - can be useful in forensic investigations [[18], [19], [20], [21]] helping to: (1) identify the author of a text, the language or the speaker; (2) establish the relationship between texts; (3) classify the type of text; and (4) characterize the profile of the author of a text. Stylometry has therefore attracted the interest of the forensic community for a number of reasons, in addition to its ability to meet the need to ’objectify’ analyses in the context of documentary forensics [22,23]. Firstly, it can be used to corroborate, or not, the results obtained by the traditional forensic examination, but also to answer questions that the latter was unable to answer. However, the use of stylometry as a forensic method in legal cases is currently very limited. Several texts report cases in which the admissibility of stylometric evidence has been questioned and rejected [19,[24], [25], [26]].

Two main steps characterize the stylometric procedure [17]: (1) the selection and the extraction of what are considered as relevant features to represent style markers, and (2) the statistical analysis of the collected data.

Information on the state of the art of stylometry is so scattered that its follow-up can result in a challenging task. Some of a large number of publications summarize the historical development of stylometry techniques [3,23,27,28]. More than a thousand features have been identified as relevant style markers [3] and countless statistical approaches have been implemented. As highlighted in the late ’90s [29], there has been no agreement either on which is best markers to select and on the statistical models for data analysis [3,30].

Stylometry is characterized by numerous challenges and developments. This is demonstrated by the existence of PAN, an organization sharing a series of information on scientific events (work-shops and conferences) and digital texts for forensic and stylometry applications. Documents are available at https://pan.webis.de.

This paper will structure information disseminated through a large scientific literature. Section 2 reports the historical development of the domain. Sections 3, 4 will focus on style markers description and selection and on the most popular statistical approaches for data analysis. Then, sections 5 and 6will present the scientific foundations of this domain and its link with forensic science with an emphasis on challenges for stylometry to guarantee the acceptance of this technical method in judicial proceedings. A conclusion will end this literature review.

## 2 The origin of stylometry and its evolution

The earliest quantitative descriptions of texts are cited by Rudman [3]. The first, dated back to 300 BCE, was made by Saunaka with reference to an ancient Indian religious text known as the Rig-Veda. Subsequently, Aristarchus of Samothrace, around 180 BCE, recorded expressions which occur either rarely or only once in Greek texts. The writer Aaron Ben-Asher, in 950 AD, counted

different elements, including the number of letters, words, and sentences appearing in the Bible. The word “stylometry” was firstly used by Wincenty Lutoslawski in his work on the chronological tracing of Plato's dialogues [31]. However, the origins of stylometry as a tool for attributing authorship to a text dated back to 1851, with the study of Augustus de Morgan [3,27,28,32], who identified word length as a good style marker. This work motivated Thomas Mendenhall to explore further the potential of this marker [23,28,[32], [33], [34]]. Mendenhall is best known for his 1901 study of the style of plays attributed to Shakespeare, where the aim was to discuss their authenticity [3,[35], [36], [37]]. Many researchers were focused on developing knowledge in stylometry; the most notorious studies have been those of George K. Zipf and George Yule.

Zipf, in 1932, showed a relationship between the number (*i*) of occurrences of *types* (*V*) and their frequency (*V*_*i*_) [23,28]. *Types* (*V*) are the total number of words in a text that do not repeat, otherwise also called the total number of different words that make up the text [15,23,38]. *V*_*i*_ is so defined as the total number of vocabulary items that are repeated *i* times. What is known as the “Zipf's First Law” [39] argues that if a list ordered by frequency of occurrence of words in a text is made, a dependency relationship is highlighted between the frequency of words and their position on the list [[40], [41], [42], [43], [44]]. Mathematically, this is described as (1):

(1) $$tfr,i=\frac{c}{r_{i}^{a}}$$

where *tf*_*r,i*_ represents the *type* frequency in *i*th rank; *c* is a normalization constant for the *Corpus*; and *r*^*α*^ the rank (*r*_*i*_) to the *α* power, where *α* was originally ∼ 1 and subsequently fitted to empirical data.

Yule, in 1938, identified the instability of sentence length as a marker, highlighting the diffi-culty of defining what a sentence is [23]. Better known is the “Yule (or K) characteristic” devel-oped in 1944;

K characteristic is a metric associated with words occurrence distribution (considered to be Poisson distributed) to describe how often words occur in a text. Many years later Gustav Herdan and Herbert S. Sichel discussed this marker and the adequacy of the Poisson distribution to model probabilistically the words’ occurrence in a text [23]. The K-value is calculated using the following formula (2):

(2) $$K=\frac{10^{4}\;\left(\Sigma_{r}r^{2}V_{r}\;-\;N\;\right)}{N^{2}}$$

where *r* represents the number of repetitions^1^; *V*_*r*_ the number of *types* that appear *r* times in the text; and *N* the total number of words in a text (*tokens*) [15,38]. The limits of applicability of the K metric were examined by Baayen [45].

In 1963, thanks to Mosteller and Wallace, an inferential method based on a Bayesian statistical multivariate approach was published [32,[46], [47], [48]]. This evaluative statistical methodology taking advantage of the Mendenhall-based marker choice was implemented to approach (probabilistically) authorship attribution on various American political texts that constitute the Federalist Papers. The choice of this marker together with the probabilistic approach has highlighted the potential offered by stylometry in the practice [23,27,28,[49], [50], [51], [52]]. Among a list of relevant features, functional words [29], including synonyms (a marker introduced by Ellegard two years earlier), were considered of extreme importance for the field. Thirty years later, this work was analysed us-ing a metric called “character *n*-grams” by Ref. [53] (a definition of character *n*-grams will be provided in Section 3), but unfortunately the probabilistic approach suggested by Mosteller and Wallace was apparently unreasonably discharged.

From 1964 until the end of the ’90s, multiple studies were carried out in order to search style markers and to develop computer-assisted textual authorship description methodologies [34]. Starting from the ’70s, technological development, accompanied by the increasing availability of software facilities and of texts in electronic format, favoured a sensible reduction in processing time [52], but also an increase in the number of published studies [23]. Subsequently, starting from the ’90s, a strong impulse to stylometric analysis was growing by the diffusion of the ma-chine learning (ML) techniques for description and classification purposes [54].

## 3 Style markers

As mentioned above, the first step of any stylometric procedure is based on the selection and extraction of stylistic features. Numerous, these features are categorized in various ways in the literature. The following is a classification of markers that attempts to take into account the various aspects presented in other studies.

### 3.1 Classification of style markers

Some features are considered as class characteristics, others are described as individual char-acteristics [13]. The first kind of features can be valuable to restrict the circle of potential authors to a specific population. They are related to external factors describing a given group, such as the social class. The second one refers to one's personal development of the language and its use; they are classified as linguistic features whose rarity is such that the probability that they could be reproduced by a third party is considered negligible.

#### 3.1.1 Lexicon

A text can be conceived as a sequence of elements, called *tokens* (words, numbers, punctuation marks) grouped in sentences [34]. Historically, the first works on stylometry were based on these elements, which are defined as style markers at the lexical level. Among the most frequent that can be listed: word length [15,23,55,56], sentence length [15,23], syllables [23,57], discourse elements [23], function and content words [23,27,28,30,58,59], and vocabulary richness^2^ [23,28,35,39,[60], [61], [62], [63], [64], [65], [66], [67], [68], [69], [70], [71]]. Savoy [41] mentions a further marker that is less common in the literature: the relative frequency of “long words” (words composed of more than 6 characters). The higher the relative frequency, the more sophisticated and complex the style.

Six main drawbacks of lexical markers have been identified: (a) the ease of fooling the system [30]; (b) the influence of editors, who can intervene and have an impact over the presence or absence of a word [30]; (c) the failure to take into account morphologically related words [30]; (d) the difficulty, or impossibility, of determining similarities concerning alternative word forms, probably due to usage errors [58]; (e) the difficulty in defining what is meant by “word” which is not necessarily easy in some languages [58,72]; (f) the dependence on the typology of the text being written, as well as on the context [23].

It's also worth noting that a different study [73] showed the importance of certain words at the level of text structure and not only with respect to frequencies and syntactic relations; their use is restricted by the length of the texts under investigation, which must be sufficiently long.

#### 3.1.2 Character

The second type of marker refers to the character level. In this case, the text is treated as a sequence of characters. More precisely, each text is represented as a frequency vector of selected character sequences. In other words, the text is thus considered to be a “bag-of-character” [74]. Historically, the study of characters in stylometry is linked to Leon Battista Alberti, whose aim was to distinguish prose from poetry in Latin works [41].

The specific character sequences chosen are called *n*-grams, where *n* represents the number of characters that make up the selected sequences. The use of *n*-grams has been introduced in the context of the characterization of poets [75]. This approach has also been used to highlight the respective contributions of several authors of a given text [46].

As this marker is independent of the language of the text,^3^ its use is particularly advantageous [17,28,34,72]. With reference to the limitation of lexical markers (*e*) mentioned above, the use of the *n*-gram is particularly interesting in cases where it is difficult to define the ‘word’, as in Asian languages (e.g., Chinese, Japanese, Korean). Furthermore, the observation of *n*-grams is also applicable in DNA sequence studies, music field [17,72], as well as for the characterization of digital code sources [76].

The use of *n*-grams is also favoured by the limited need to pre-process the text^4^ [80] and by the fact that they can be easily extracted and counted [58]. Furthermore, another advantage is the small impact of noise, possibly caused by language mistakes or misprints [58].

Since altering a style at the character level is more difficult than at the lexical level, the use of this marker is to be preferred in cases where there is a suspicion of active malice [28], notably a deliberate alteration of writing style for malicious purposes.

Leaving aside the ease of extracting this type of marker [28], its implementation in the practice is hampered by two concrete difficulties: the choice of the character sequence length (*n*) and the length of the text profile size (*L*). By definition [80]:

A profile *P* is a set of *L* pairs (*g*_1_*, f*_1_)*,* (*g*_2_*, f*_2_)*,* …*,* (*g*_*L*_*, f*_*L*_), where *g*_1_*, g*_2_*,* …*, g*_*L*_ are the *L* most frequent n-grams of the text (in decreasing order) and *f*_1_*, f*_2_*,* …*, f*_*L*_ their normalized (wrt text length) frequencies of occurrence, respectively (at p.237),

and the optimal character sequence length (*n*) was found to be language dependent, as the average word length can vary [17,28,46]. In the literature there can also find proposals according to which a combination of, say, 2-g, 3-g or even more is to be preferred to a single value for *n* [17,28,46]. Clearly, this leads to an increase in dimensionality requiring features reduction methods. Another approach would be to take into account only the dominant *n*-grams as suggested by Houvardas and Stamatatos [46]:

The main idea is to compare each n-gram with similar n-grams (either longer or shorter) and keep the dominant n-grams. (at p.79)

#### 3.1.3 SYNTACTIC elements

The idea that every author uses, unconsciously, the same syntactic pattern leads to the identifi-cation of this third category of markers [34]. Texts can, for example, be broken down into what is called “Part-of-speech” (POS) and then the different parts can be used to search for features such as punctuation [30]. The latter marker is considered very effective in cases where texts have not been subjected to any edited review [77].

Syntactic information as a marker has two important limitations: it is language-dependent and highly vulnerable to analysis errors [28,34]. Despite the fact that syntactic structure can be characterized by high intra-variability [81], it has more reliability than lexical markers [82].

#### 3.1.4 Semantic elements

The study of semantics consists in analysing the context in which words are used. For example, it has been said that in Corneille's comedies, the word “love” is associated with the father figure [41]. As elements that reflect the meaning of texts, semantic elements include subjects [83].

While the markers previously described can be automatically investigated, the complexity of semantic analysis makes its application for stylometric analyses difficult to implement [34].

#### 3.1.5 Specific features

Application-specific features of text, like its structure and layout, anomalies and metadata (i.e. information describing electronic documents) [30], can also be used as stylometric markers. They define the structure of a text and are particularly useful when analysing email texts and web forum discussions [34].

Neal et al. [28] also distinguished so-called “content-specific” features of the text, which in-clude keywords and other information about the topic of the document.

#### 3.1.6 Other textual elements

Faults or words that reflect a social or cultural reality has been suggested as additional markers [28]. Note the difficulty in their classification into one of the above-mentioned typologies of markers.

### 3.2 Some remarks

As pointed out by Athira and Thampi [84], the choice of the style marker to be targeted is a fundamental step in text analysis. However, to this day, there is no consensus on which marker should be considered as the best. Traditionally, the choice of stylometric marker was left to the scientist's personal choice [85].

In 2007, a study focused on comparing the effectiveness of 39 style markers was published [15]. The *n*-grams were the markers that achieved the highest accuracy rates. Overall, a de-crease in accuracy could be observed as the number of authors in the *Corpus* (i.e. the set of reference/comparison material) increases. The use of 2-g and 3-g provided the best re-sults. As reported by Grieve [15], previous studies have shown the effectiveness of longer *n*-gram. Grieve points out that this effectiveness may be related to the fact that all analysed texts in previous studies had the same common topic and so longer *n*-grams are therefore more discriminating.

The use of the most frequent words and *n*-grams as markers have provided the most promising results in recent scientific works [86]. As reported by Koppel et al. [51], *n*-grams perform well in the forensic context and have been defined as very sensitive and accurate markers [87]. The choice of selecting and excluding the most frequent words as markers is proposed under the assumption that there are classes of words beyond the author's control, whereas *n*-grams have shown language independence and the possibility of capturing different stylistic information,^5^ favouring their use [54].

Stamatatos [58] makes the reader aware that studies are often conducted using extended texts characterized by similarities in the genre and topic. This does not correspond to practical (case-related) conditions observed in forensic science, where the available questioned and reference materials are often extremely limited.

The author points out that controlled conditions in scientific studies inevitably lead to an over-estimation of the effectiveness of the method. By taking texts of different genres and topics, Sta-matatos [58] was able to report the effectiveness of the *n*-grams in comparison to function words (e.g. prepositions, common adverbs, pronouns and articles [59]) and their accuracy.

Style markers can also be used in combination [34,84]. The joint consideration of features leads to an increased amount of information [30], but also in dimensionality, which can have noisy effects affecting parameters estimation procedures due to recurrent characteristics [34,41].

## 4 Data analysis

By the ’90s, multivariate statistical analysis to depict groups of writers was used as an alterna-tive to previous univariate approaches based on the measurement of a single feature [23,30,82,88,89].

It is not the purpose of this paper to exhaustively review and summarize all statistical tech-niques used in the stylometric field. Below, the reader can find a general overview of the most cited methods in the literature [3,23,28,30,35].

A long list of techniques for descriptive and inferential statistical approaches were described for stylometric data analysis since late ’60. These techniques refer to Ref. [23]: chi-square test (see, e.g. the works of Brinegar [90]), factor analysis (e.g. Miles and Selvin in 1966), discriminant analysis (e.g. Cox and Brandwood in 1959, Somers in 1966, Bruno in 1974 and Ledger in 1989) and clustering (e.g. Bailey in 1979, Boreland and Galloway in 1979, Ledger in 1989 and Holmes in 1992). The general aim of those proposals is to describe and to detect similarities or dissimilarities between features of relevant markers in different texts.

A criticality that characterized this type of application is linked to the high dimensionality of the available data. Principal component analysis (PCA) was recognized as a good method in the field of stylometry [11] and indeed it has proven to be a viable alternative for dimensionality reduction [30]. Historically, some of the most significant studies relying on this method include the works of Burrows and Hassall, 1988; Burrows, 1992; Smith, 1991 and Holmes, 1992 [23]. PCA was also used to show that the 15th Oz book’ writing style is more compatible with that of Thompson's than that of Baum, author of the earlier Oz books [31]. Note that some criticisms on the use of PCA in genetics studies have been published recently [91].^6^

In the ’90s, some studies based on correspondence analyses (a multivariate technique used to describe potential relationships through graphical representations [92]) has been published. In the same years, A. Q. Morton introduced into the field of textual authorship studies the so-called cumulative sum control chart (CUSUM), a statistical technique that quantifies the cumulative sums of deviations of the sample values from a target value [32,93]. The validity of this proposal has been seriously questioned by several researchers, mainly because of a lack of clarity both in the interpretation of results and in the understanding assumptions [29,94].

As mentioned at the end of section 2, machine learning (ML) techniques play an important role in stylometry [11,16,28,35,49,53,58,75,[95], [96], [97], [98]]. These techniques provide higher accuracy than descriptive statistical methods, as well as better tolerance to noise and non-linear interactions between different style markers [82]. Some critical remarks on their use have been highlighted by Ref. [99]:

Once you unleash it on large data, deep learning has its own dynamics, it does its own repair and its own optimization, and it gives you the right results most of the time. But when it doesn’t, you don’t have a clue about what went wrong and what should be fixed. In particular, you do not know if the fault is in the program, in the method, or because things have changed in the environment. We should be aiming at a different kind of transparency. (at p. 1)

Dimensionality reduction and classification are the main advantage of ML techniques, regard-less of whether they are used in supervised or unsupervised learning environments. However, as shown in earlier studies, the dimensionality of forensic data does not represent a barrier (see, e.g., Refs. [100,101]). As pointed out by Biedermann [102], there are at least two major problems that the implementation of a ML-based approach poses with regard to forensic identification problems, such as the authorship procedure in stylometry. Primary, it is important to recall the purpose of the forensic evaluative process and in particular the role of the expert in the authorship attribution. Secondly, a ML-based approach provides inferential answers based solely on the data used to build the model. What about case-related information and their role in the process? As noticed by, e.g., the Swiss jurisprudence,^7^ the expert assess the value of data so to provide a statistical information and it is not superfluous to assess all other evidence in order to answer a question of legal interest and deliver a final conclusion on the hypothesis of interest (i.e., authorship). Moreover, decision theory plays an important role in legal procedures; in fact, a decision maker should coherently justify their conclusion and to do so he should adopt a decision theoretical point of view where in addition to the uncertainty that characterizes the elements of decision-making, there is also the need to specify a preference ordering among the consequences associated with each decision and quantify their undesirability [103,104]. Biedermann [105] criticized the use of ML in forensic science:

[t]he persistence of source attribution claims [individualization or authorship] in foren- sic science literature is problematic. […] Machine learning not only bears potential for misapplication for the purpose of forensic source attribution, but that such misap- plications actually do occur. (at p.1)

In many scientific or industrial sectors, ML technique can be thought of as an engineering tech-nique that provides valuable tools for optimization, but its use in forensic science is still questioned.

The authorship process requires transparency and robustness in the model and the rational use of contextual case information. The term “transparency” (in the words of Jackson [106])

[explains] in a clear and explicit way what we have done, why we have done it and how we have arrived at our conclusion. We need to expose the reasoning, the rationale, behind our work. (at p. 84)

The term “robustness” challenges a scientist's ability to explain the ground for their opinion to-gether with their degree of understanding of the particular evidence type. Do the current inferential statistical methods used in practice satisfy those requirements?

Other descriptive methods based on distance or similarity [85] between given texts are also widely implemented to deal with textual authorship attribution [28,30,107,108] but with a ques-tionable line of reasoning: the smaller the distance value, the higher the probability that the texts were written by a given person (author) [38,78,108].

It is worth noting that a forensic scientist should evaluate the available data (e.g. data summarized by similarity scores or distances between features extracted from questioned and reference texts). Once the distance between the questioned texts has been calculated (or a list of features described), the expert should (as requested by the ENFSI guideline for evaluative reporting [109]) assign (at least) two conditional probabilities: the probability of obtaining such measurement un-der the hypothesis that the texts were authored by the same source (author) from one side, and under the hypothesis that the texts were written by different authors, from the other side.

Evaluation will follow the principles outlined in Guidance note 1 (refer to paragraph 4.0). It is based on the assignment of a likelihood ratio. Reporting practice should con- form to these logical principles. This framework for evaluative reporting applies to all forensic science disciplines. The likelihood ratio measures the strength of support the findings provide to discriminate between propositions of interest. It is scientifically accepted, providing a logically defensible way to deal with inferential reasoning. Other methods (e.g., chemometrical methods) have a place in forensic science, to help answer other questions at different points of the forensic process (e.g., validation of analytical methods, classification/discrimination of substances for investigative or technical reporting). Equally, other methods (e.g., Student’s t-test) may contribute to evaluative reports, but they should be used only to characterize the findings and not to assess their strength. Forensic findings as such need to be distinguished from their evaluation in the context of the case. For the latter evaluative part only a likelihood ratio based approach is considered. [109], p. 6

The ratio between these two probabilities is known as the likelihood ratio, or more generally the Bayes factor, the coherent measure for the value of the evidence [110]. In stylometry, this probabilistic approach is rarely implemented or discussed. A partial probabilistic model, referring just to one of the two hypotheses, has been proposed by Ref. [79]. A Bayesian probabilistic approach has been recently proposed in stylometry by Ref. [111] in the context of authorship discrimination between French authors of classical plays.

## 5 Forensic science and stylometry

Following the themes presented in the previous sections, it is not surprising that forensic science can show an interest in stylometry. Before delving more deeply into the link between the two disciplines, it is necessary to elaborate on the concept of ‘textual authorship attribution’. This notion is extended to four principal different situations: identification, verification, profiling and similarity detection.

A problem of authorship attribution may be based on a question of identification or verification. In the first case, the unknown identity of the author is sought. In the second case, the textual origin of different documents is analysed in order to determine whether or not the texts come from the same source [28,71,112]. Generally, in the case of verification the questioned text is compared only with reference texts sourced by a given candidate [41,51]. Identification and verification questions can also be treated as classification cases [113], which in turn can be described as: (i) a binary classification where all the questioned documents will be considered to have been written by one of the two known authors: (ii) a multi-class classification where all the questioned documents come from more than two known authors and; (iii) finally, a one-class classification where there is only one known author and the aim is to determine which document(s) was (were) written by that author [2,12,34,114].^8^

Profiling aims to help determine the personal, demographic or psychological characteristics of the author(s) of a text. Typical questions encountered may concern: gender, age, social class, education level, and nationality of the author(s), as well as the number of individuals behind the writing of a text [30,51,71,108,115,116].

Vice-versa, in the so-called “similarity detection” situations, the scientist's interest is to investigate whether texts were written by a single author without ascertaining their identity and/or inferring their personal characteristics. This is typically what is sought in suspected plagiarism cases [82]. While the software currently implemented in practice has the ability to detect direct plagiarism (copy/paste of each word), the problem is more difficult in the case of so-called “the-saurus plagiarism” characterized by the use of synonyms and a mixture of sentences from different texts. Moreover, difficulties arise when a third party wrote a text by officially substituting their mandating party [117].

Other situations were described by Savoy [41] who further distinguishes between ‘author clus-tering’ and ‘author linking’ problems. In the first case, the interest is in detecting the number of authors hidden behind a sample of *n* texts, whereas in the second case one is interested in deter-mining the texts that were written by the same person, while not having any information on the number of writers.

However, it should be emphasized that stylometry is not focused exclusively on problems of authorship attribution [118]. Another branch of stylometry, known by the term “stylochronometry”, focuses on problems of a chronological nature (e.g. dating a text) [28,29,35].

Stylometry can play a valuable contribution in providing answers of great interest for the foren-sic field [4], say, did the suspect actually write the text in question? Who, among a group of people, could have written the text in question? Were these documents all written by the same person? When was the text written? What is the gender (or, e.g. the mental state) of the person who wrote the text?

These questions may be encountered on various scenarios; in fact, the applicability of sty-lometric methods is not limited to issues of pseudonymity, plagiarism - professional, literary (especially with the creation of e-books) or musical - or contractual cheating. Other areas of interest that may be subjected to stylometric analysis are included in the non-exhaustive list below: employment contracts, threatening or harassing letters, wills, testimonies, extortion at-tempts, threats of attack (e.g. related to terrorism), suicide letters, malware, cyber harassment, sms messages, emails, blogs (e.g. in the field of child pornography) or, again, defamatory posts [1,20,21,24,28,34,46,74,79,82,84,87,114,117,119].

Stylometry applied to the above-mentioned scenarios may lead to discussions at trials around its admissibility as reliable technique.

### 5.1 The theoretical foundations of stylometry

If we consider the definition of stylometry as an approach to infer the characteristics or the identity of the author of a text based on discriminating stylistic features [16], the importance of so-called features, otherwise referred to as stylometric markers, is evident. Their main property is that these are typically chosen unconsciously by the writer [17]. More than a few properties were mentioned in Ref. [29]. It is reported that:

[T]hey should be salient, structural, frequent and easily quantifiable, and relatively immune from conscious control. By measuring and counting these features, stylometrists hope to uncover the ’characteristics' of an author. (at p. 111)

Textual attribution follows from the basic assumptions of stylometry, notably that (a) each individ-ual has a single, verifiable style [3] (therefore two individuals can be discriminated by their style) and that (b) one's style evolves over time [2], as a result of the writer's personal development [98], but it is still discriminating.

Since 1964, styles were defined as deviations from a norm [120]. Unconsciously, each indi-vidual uses a unique way of writing text, which is described by quantifiable markers that allow for a style characterization [27,98,121]. Here is an excerpt from McMenamin's statement in the *Ceglia vs Zuckerberg* expert opinion [122], which sums up the concept:

Individual differences in writing style are also very often due to an individual’s choice of available alternatives within a large, shared common pool of linguistic forms. At any given moment, a writer picks and chooses just those elements of language that will best communicate what he/she wants to say. The writer’s ’choice’ of available alternate forms is often determined by external conditions and then becomes the unconscious result of habitually using one form instead of another. Individuality in writing style results from a given writer’s own unique set of habitual linguistic choices. (at p. 3)

As a result of the above, the assumption concerning the evolution of a person's style over time seems to be plausible. In addition to the effect that certain diseases, such as dementia, may have on writing style, a decrease in the diversity of word usage (also referred to as richness of style) has been observed when considering a writer's style over time [23,123,124]. In this regard [41], explains that 5 life phases can be associated with the language and style of each individual: the infant phase, the child phase, the adolescent phase, the adult phase and the elderly phase. In the adult phase the style undergoes little variation and is relatively stable.

The study by Ref. [124] was able to show the possibility of classifying a text correctly within an interval of time. The authors pointed out, however, that longer periods of time between the writing of two texts do not necessarily imply glaring changes in style.

Although style is unique to each individual, different styles can be adopted by the same person (according to background, text genre, writing period, theme, text form, or audience [113,125]). This implies that there is some variability in personal style, in addition to the variability that might be due to the passage of time. These two aspects recall the two fundamental “laws” of handwriting [126]: the first referring to the inter-variability (no two individuals write exactly alike) and the second relating to the intra-variability (no one person writes the same word the same way twice) of handwriting.

Analogous to cases typically encountered in forensic science, the problem of textual author-ship is addressed by searching for a “stylistic fingerprint” of the questioned text in order to make a comparison with the stylistic fingerprint characterizing the reference material [78]. The set of comparison material is referred to as the *corpus*. Basically, the questioned text is thus compared with texts originating from a given population, which will be specified on the basis of the hypoth-esis set in question [3]. Closed-class scenario refers to the situation where it is assumed that the true author is one of those whose texts make up the *corpus* [71,79,88]. In contrast, the open-class problem admits the possibility that a third party, not belonging to the *corpus*, may be the author of the questioned work [30,127] such as in the “Ferrante case” studied by Mikros [54].

Some publications [3,79] attempt to list the conditions necessary to ensure quality comparison material. Its textual authorship must certainly be known and verified. Following the assumption that time influences the style of each individual, reference material should be as contemporary as possible with the questioned text. Other assumptions, including the dependency of style or literary genres/types of text need to be tested further, though it seems that this may have a negligible impact when certain stylometric markers are used [98].

What must also be tested is the minimum length that the questioned text should have in order to guarantee robust results, as well as the minimum amount of reference material. This question is often mentioned in the literature and represents a real practical problem. Nowadays, with the increasing use of portable media and devices, stylometry is increasingly striving to be applied to shorter and shorter texts. Obviously, in contrast to research studies, real forensic cases are characterized by short texts and a very limited amount of material [16,34,47,58,116,[128], [129], [130]].

### 5.2 Admissibility

The admissibility of so-called *language evidence* in court was discussed in the early 2000s, notably in *United States vs Van Wyk* [128]. At the heart of the still limited use of stylometry as a scientific methodology to effectively address forensic cases is the lack of scientific studies on the effectiveness and robustness of the methods employed [79,131]. Chaski [128] explains:

The Defence argued that the ‘proffered expert testimony is subjective, unreliable and lacks measurable standards’ (Van Wyk, 83 F.Supp.2d 515, 521). Thus, the Defence argued that admitting forensic stylistics testimony would violate several other criteria of the Daubert standard of empirical reliability, such as falsifiability of the technique, known error rate, and standard operating procedures for performing the technique (see Note 2 for more discussion of admissibility factors). (at p. 2)

In fact, one should guarantee that the technique has been (or could be) tested, that the technique has been published in peer-reviews, that the technique is accepted by the scientific community and that its error rate is known [[132], [133], [134]]. A further fundamental aspect relates to the adequacy of the evaluative procedure and the adherence to the published international standards (see, e.g. recom-mendations offered by the ENFSI guidelines [109]) to deal with the assessment of the available descriptive data of a series of texts.

The aspects that mostly limit the use and admissibility of stylometry for forensic purposes are method credibility and potential active malice [30]. The term “credibility” hides different requirements [30]; in order to be credible a method must be accurate and the degree of accuracy must be determined, its scientific foundations must be recognized, and finally the full methodology must be transparent and robust. While this is partially reported in the literature, little is known about the deliberate alteration of writing style for malicious purposes (adversarial stylometry). Three alteration techniques are known: imitation, translation and obfuscation [28]. Savoy [41] points out that imitation is a very difficult task both to accomplish and to detect. The techniques of translation and obfuscation are less complex. The first practice consists of translating the original text into one or more languages and then returning to the original language, while the second one is characterized by the substitution of words following the identification of the stylistic profile that one wishes to change.

Thus, although stylometry has existed for centuries and its use is scientifically documented, its implementation as a scientific method to effectively deal with court cases is still discussed and rather limited.

## 6 Conclusions

The principal aim of this paper was to conduct a review of the state of the art of stylometry, along with a description of the main historical stages, and most importantly a discussion focused on the link with forensic science, its potential contribution and the eligibility of these tools in court. The persistence of critical issues that must be addressed to allow (or favour) the implementation of these tools in the forensic field is palpable. Open issues include technical and evaluative aspects. There must be included the choice of the marker, the choice of the distance to quantify similarities, or the minimum requirements in terms of quantity and quality of material. However, there are still major gaps to be filled, particularly with regard to the evaluation of the measures collected and for decision-making purposes such as identifying an author, characterizing the author, detecting a false document, detecting plagiarism, the contribution of several authors, etc. Ishihara [135] had already pointed out the failure of forensic stylometry that unlike other forensic fields (including handwriting examination) does not promote (or support) the use of a likelihood ratio for evaluative and investigative issues of forensic interest.

The probabilistic approach proposed by Bozza et al. [111,136] represents a first attempt in this direction. Champod et al. [137], as reported by Riva [138], highlighted the problematic issue of probability assignment. Unfortunately, this aspect is still wrongly considered to be a major obstacle to the wider use of the probabilistic approach.

It must be pointed out that, from the review of the state of the art in the field of stylometry, it emerges quite clearly that the role of the scientist in rarely respected. The scientist is asked to evaluate observations made on the material under examination and on reference materials. This definition is found in many legal texts. The role of the scientist is to evaluate observations and not hypotheses that may be of interest to the Court [139,140]. To fulfil the meaning of terms such as “identification”, “verification” and “profiling” (as usually mentioned in stylometry), it is not sufficient to consider only data collected on available materials. As stated above throughout law jurisprudence, any information gathered during the investigative procedure related to the disputed document is of essential interest to justify a decision about the hypotheses. In other words, the expert must focus exclusively on the value of the observation (i.e. the data obtained through stylometry analysis), and not on the evaluation of the hypotheses (i.e. the text was written by author X). Thinking that an *ad hoc* statistical method (i.e. ML technique, PCA, etc.) solely based on data can solve the legal problem and allow one to express their opinion on an hypothesis is inappropriate. Each actor involved in the criminal proceeding should respect their role. The scientist must be able to quantify the value of the evidence and provide this information to the judge, so that it can be integrated with the mass of information available to the Court in order to reach the final verdict. Note that this should include the quantification of the undesirability of decision outcomes (e.g., a false authorship attribution). Needless to say, the full Bayesian model is the only one that allows one to perform this sequence of evaluation and decisions.

## CRediT authorship contribution statement

**Valentina Cammarota:** Writing – original draft, Conceptualization. **Silvia Bozza:** Writing – review & editing, Validation. **Claude-Alain Roten:** Conceptualization. **Franco Taroni:** Writing – review & editing, Validation, Supervision.

## Declaration of competing interest

The authors declare that they have no competing financial interests or personal relationships that could have appeared to influence the work reported in this paper.

## Footnotes

1 Please the reader may be careful: the meaning of *r* in the two (1), (2) is not the same.

2 The vocabulary richness is not necessarily very effective when it comes to problems of authorship, but it is helpful in cases where one wishes to distinguish the writings of a machine from those produced by a human being [41].

3 In order to avoid misunderstandings, it should be made clear that the *n*-gram model is language-independent. In the sense that no language-dependent adaptations are necessary. Regardless of the language, this marker can be used. However, all texts in the same case must be written in the same language.

4 It is important to note that the published version is subject to the intervention of editors and others [30], which could lead to changes in the original text [77,78]. Compared to the holograph, the first edition remains the clos-est [3]. Pre-processing is often necessary before the analysis of texts to remove elements that may belong to the reviewers/editors [79].

5 Characters markers provide lexical, syntactic and/or structural information [46,80].

6 It has been stated that “Principal Component Analyses (PCA)-based findings in population genetic studies are highly biased and must be reevaluated.”, and that “We conclude that PCA may have a biasing role in genetic investi-gations and that 32,000–216,000 genetic studies should be reevaluated.” [91].

7 See, e.g. Bulletin de Jurisprudence Pénale, 4 (1997) 103–105.

8 It is legitimate to estimate that this categorization is not exhaustive, in fact, it lacks, for instance, the case where several authors may be the source of a text.
